# Supplementary material for: Generation of Peptides for Highly Efficient Proximity Utilizing Site-Specific Biotinylation in Cells
Source: Life (Basel). 2022 Feb 16;12(2):300. doi: 10.3390/life12020300 (PMC8875956; doi:10.3390/life12020300)
Supplement: Supplementary file 1 [file life-12-00300-s001.zip › Supplementary data.pdf]

WESTERN BLOTTING PROTOCOL (TIMING 2 d)

Materials

NuPAGE 4–12% (wt/vol) Bis–Tris SDS-PAGE precast gel (Invitrogen, #NP0336), NuPAGE LDS sample buffer (4×) (Invitrogen, # NP0007), DTT (Sigma-Aldrich, # D9779), UltraPure Tris (Thermo Fisher Scientific, # 15504020), Glycine (Merck Millipore, # 357002, CAS: 56-40-6), 100% (vol/vol) Ethanol (Sigma-Aldrich, # E7023), MOPS-SDS buffer (20X, Thermo Fisher, # NP0001), SDS (Thermo Fisher Scientific, # 15525017, CAS: 151-21-3), Spectra™ Multicolor Broad Range Protein Ladder (Thermo Fisher, # 26634), Tween-20 (Sigma-Aldrich, #P9416-100ML), Nonfat dried milk powder (AppliChem, # A0830,1000), Hybond™-C nitrocellulose membrane (Amersham Biosciences, # RPN203D), HRP-conjugated streptavidin (Invitrogen, # 43-4323), Penta His HRP conjugated Anti-polyHistidine antibody (Qiagen, # 34460), Cheluminate-HRP PicoDetect, solution A (AppliChem, #A3417,1200A), solution B (AppliChem. # A3417,1200B), Amersham Hyperfilm™ ECL (GE Healthcare, # 28906837).

Reagents setup

Transfer buffer. 25 mM Tris, 192 mM glycine, 10% (V/V) ethanol, 0.1% (W/V) SDS.

Equipment

Mini-Cell Electrophoresis System (Invitrogen), Wet/Tank Blotting Systems (Bio-Rad), Heidolph Duomax 1030 Rocking Platform Shaker.

Procedure

1. 5µl of LDS sample buffer (4×), containing 50 mM DTT was added to the 15 µl of samples and mixed slowly by pipetting.
2. Tubes were boiled 5 min at 95 °C and spun on minicentrifuge at maximal speed.
3. Two NuPAGE 4–12% (wt/vol) Bis–Tris SDS-PAGE precast gels were prepared, one of which was used for His-tag detection and the other for biotin detection. Combs from gel cartridge were removed; the lanes were rinsed by MOPS-SDS buffer and the cartridge was placed to Mini-Cell Electrophoresis System.
4. Equal amount of each sample were loaded to two cassettes (10 µl), camera was filled with MOPS-SDS buffer and run at a voltage of 100 volts for 120 minutes.▲**CRITICAL STEP** during loading sample it is recommended to avoid bubble formation and take less volume 8-9 µl of the sample per lane to keep equal amount for both gel cartridges.
5. The gel cartridges were opened carefully; the gel was transferred on nitrocellulose membrane and covered by filter papers soaked with transfer buffer and placed in cassettes. Cassettes were locked and placed into transfer tank filled with transfer buffer and run at a voltage of 100 volts and a current of 350 mA for 110 minutes.
6. Membranes were blocked using a solution containing PBS with 0.2% Tween and 5% milk (stirred on the platform shaker 30 min at room temperature). ●**PAUSE POINT** Membranes can be stored ON or 2-3 days at +4 °C.

7. Membranes were washed 10 min three times with 15 ml of PBS solution with 0.2% Tween.

8. Membranes were incubated in 10 ml of PBS with 0.2% Tween, 0.125% milk and 2 µl streptavidin-HRP or anti-His-HRP on the shaker for 45 minutes.

9. Membranes were washed 15 min three times with 10-15 ml of PBS solution with 0.2% Tween.

10. Membrane were treated with a mixture of 500 µl Luminol / enhancer solution and 500 µl Stable peroxide buffers (Applichem, A3417, 1200), transferred in a thin transparent polyethylene film and placed in a cassette with an autoradiography film (in the dark) to detect the signals of tagged proteins. To obtain a distinct picture, a film was held on the membrane with a threefold increase in the exposure time (5, 15, 45, and 135 seconds).

11. Film was developped on a developing machine.

Table S1. *Troubleshooting table.*

Step	Problem	Possible reason	Solution
Step 4	Bubbles in sample	Fast pipetting during loading	Repeat step 2
Step 11	No signal	The protein of interest is not abundantly present in the sample	Repeat transfection using fresh cells (reagents, kits) or use an enrichment step to maximize the signal (e.g. purify His-tagged proteins from lysates)
		The detection kit is old and the substrate is inactive	Use fresh substrate or add 20 ml of 3% H_2O_2 to 500 ml of Cheluminate-HRP PicoDetect solution B
Step 11	High background	Blocking of non-specific binding might be insufficient	Increase the blocking incubation period or concentration of milk in the antibody buffers. Consider changing milk to the blocking agent from the Penta His HRP Conjugate kit
Step 11	High level of background biotinylation in control experiment	Endogenous biotin present in FBS	Dialyze FBS
Step 11	Low transfection efficiency	Improper pH or storage of 2×HBS	The optimal pH of the 2×HBS solution should be determined experimentally. The 2×HBS solution should be stored at -20 °C
		Status of HEK293T cells	HEK293T cells should not be used 18-20 passages and cells should not be allowed to become overconfluent. Cells should be split twice a week at a 1:12 ratio
		Plasmid DNA quality	Use endotoxin free high purity plasmid with A260/280>1.80
