# Supplementary material for: Generation of Peptides for Highly Efficient Proximity Utilizing Site-Specific Biotinylation in Cells
Source: Life (Basel). 2022 Feb 16;12(2):300. doi: 10.3390/life12020300 (PMC8875956; doi:10.3390/life12020300)
Supplement: Supplementary file 1 [file life-12-00300-s001.zip › Supplementary Figures S1 and S2.pdf]

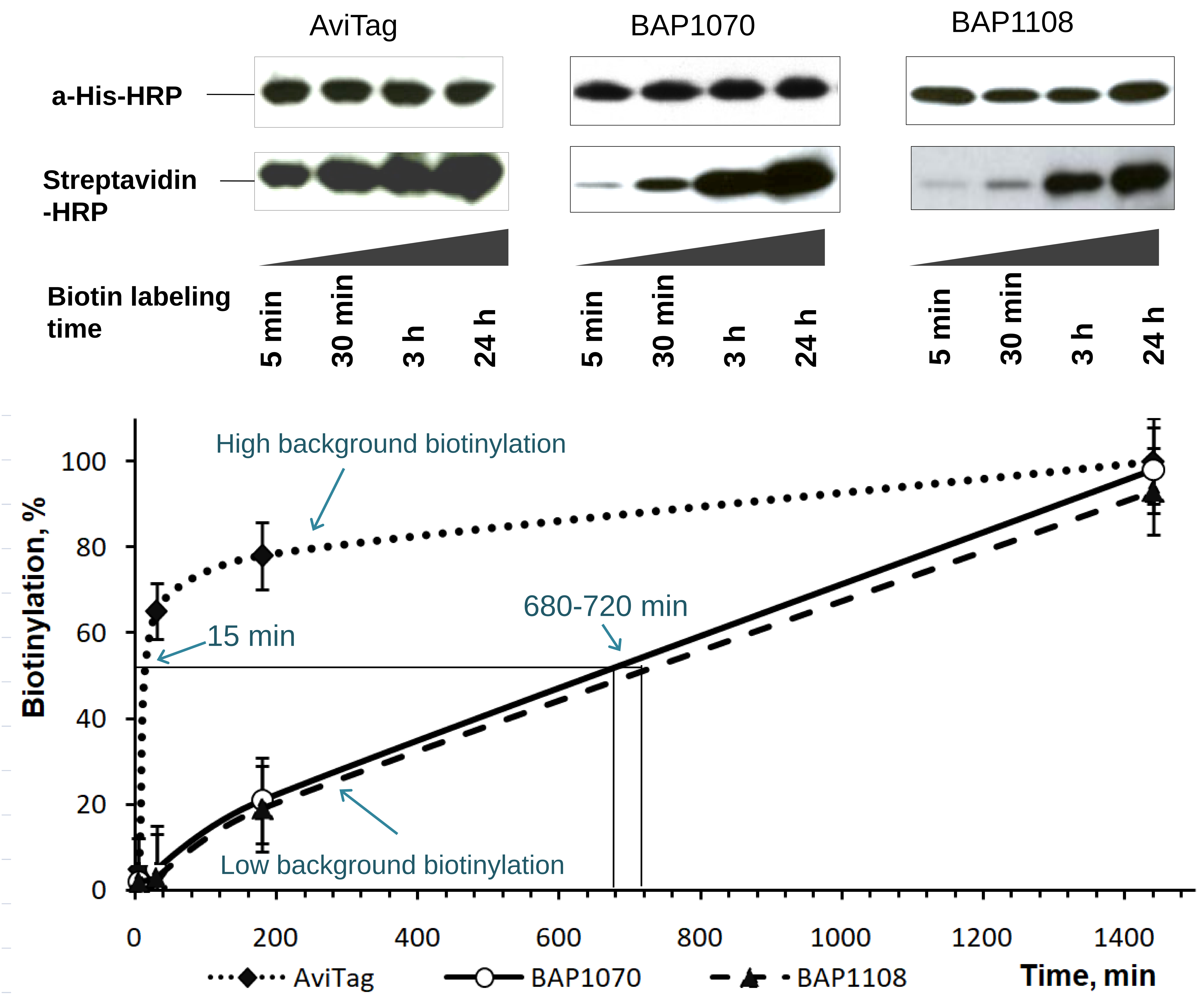

Figure S1. Densitometric quantification of the kinetics of biotinylation of the BAP1070 and BAP1108 versus AviTag (MAGLNDIFEAQKIEWHE) in the model experiment BirA-GFP+BAP-GFP. The streptavidin signals were recalculated after normalization of α-7×His signals. For each individual experiment, the normalized biotinylation level for each point was divided by the corresponding value for the 24 hour point from the same experiment, which was taken as 100%. Plotted are averaged results from three independent experiments.

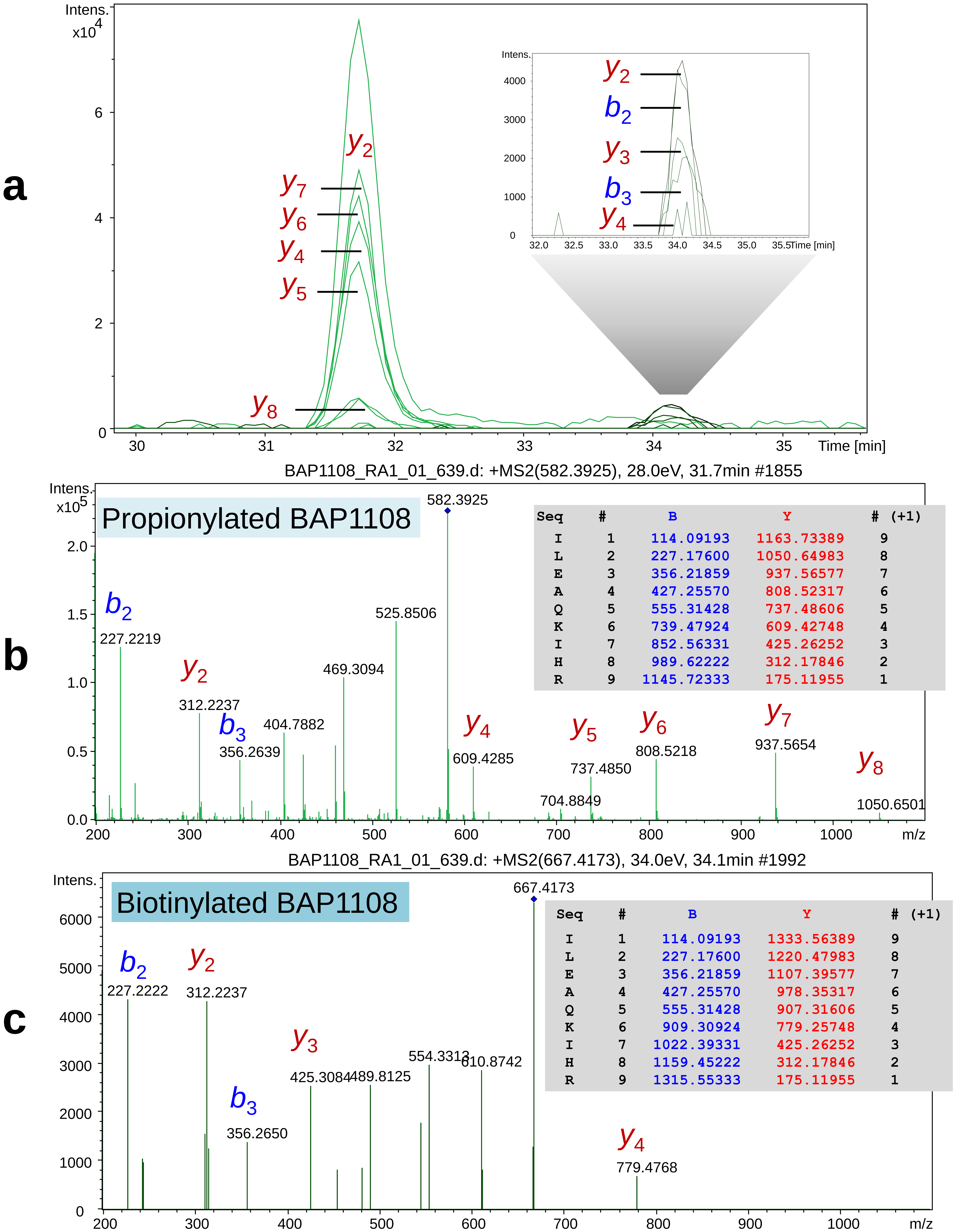

Figure S2. Detection of propionylated and biotinylated forms of BAP1108 peptide in the model experiment BirA-GFP+BAP-GFP. Extracted ion chromatograms (**a**) for the most intensive fragmentation ions present in the MS/MS spectra of the propionylated BAP1108 (**b**) and biotinylated BAP1108 (**c**) peptides are shown. The data were obtained on Thermo Dionex Ultimate 3000 nano HPLC system connected via CaptiveSpray to QTOF Impact II mass spectrometer. The predicted m/z values (highlighted in grey) of the fragment ions were calculated using a fragment ion calculator (http://db.systemsbiology.net/proteomicsToolkit/FragIonServlet.html).
